# Supplementary material for: Interaction between Metabolic Genetic Risk Score and Dietary Fatty Acid Intake on Central Obesity in a Ghanaian Population
Source: Nutrients. 2020 Jun 27;12(7):1906. doi: 10.3390/nu12071906 (PMC7400498; doi:10.3390/nu12071906)
Supplement: Supplementary file 1 [file nutrients-12-01906-s001.pdf]

Supplementary Materials

Table S1. Genotype distribution of the fifteen SNPs that were included in the metabolic-GRS

Gene	SNP	Genotype	Genotype Frequency	Nucleotide change	MAF	dbSNP* frequency in Africans	HWE
TCF7L2	rs12255372	GG	117	G/T	T= 0.37	G=0.73	0.47
		TG	144			T=0.27	
		TT	37				
TCF7L2	rs7903146	CC	162	C/T	T= 0.27	C=0.74	0.42
		TC	111			T=0.26	
		TT	24				
MC4R	rs17782313	CC	19	C/T	C= 0.25	T=0.66	0.86
		TC	110			C=0.34	
		TT	168				
MC4R	rs2229616	GA	9	A/G	A= 0.02	G=0.99	0.79
		GG	290			A=0.01	
PPAR	rs1801282	CC	298	C/G	G=0	C=1	0.98
		GC	1			G=NONE	
FTO	rs9939609	AA	60	A/T	A=0.47	A=0.52	0.11
		TA	163			T=0.48	
		TT	76				
FTO	rs10163409	AA	296	A/T	T=0	A=0.98	0.95
		TA	2			T=0.02	
CDKN2B	rs10811661	CT	18	C/T	C= 0	T=0.98	0.59
		TT	280			C=0.02	
KCNQ1	rs2237895	AA	230	A/C	C=0.12	A=0.85	0.02
		CA	69			C=0.15	
KCNQ1	rs2237892	CC	203	C/T	T=0.16	C=0.9	0.09
		TC	90			T=0.1	
		TT	4				
ADIPOQ	rs266729	CC	248	C/G	G= 0.08	C=0.92	0.12
		GC	49			G=0.08	
ADIPOQ	rs17846866	TT	297	G/T	G=0.0	T=1	-
						G=0	
CAPN10	rs2975760	CC	1	C/T	C= 0.02	T=0.7	0.01
		TC	10			C=0.3	
		TT	281				
CAPN10	rs5030952	CC	57	C/T	C=0.44	T=0.53	0.90
		TC	145			C=0.47	
		TT	95				
CAPN10	rs3792267	AA	1	A/G	A= 0.12	G=0.88	0.09
		GA	67			A=0.12	
		GG	227				

Abbreviations: SNP, Single nucleotide polymorphisms; GRS, Genetic risk score; MAF, Minor allele frequency; HWE, Hardy- Weinberg equilibrium; *TCF7L2,* Transcription factor 7-like 2; *MC4R,* Melanocortin 4 Receptor; *FTO,* Fat mass and obesity-associated; *ADIPOQ,* Adiponectin; *KCNQ1,* Potassium voltage-gated channel subfamily Q member 1; *CDKN2A/2B,* Cyclin dependent kinase inhibitor 2A/2B; *CAPN10,* Calpain 10; *PPARG,* Peroxisome proliferator-activated receptor gamma.

* dbSNP database: https://www.ncbi.nlm.nih.gov/snp/

Table S2. Characteristics of the study participants stratified based on sex.

	Total (N=302)	Men (N=126)	Women (N=176)	P value *
Age (years)	38.17 ± 9.64	35.97 ± 9.02	39.74 ± 9.79	<0.001
BMI (kg/m2)	26.63 ± 4.99	23.63 ± 3.12	28.79 ± 4.96	<0.001
WC (cm)	88.48 ± 12.41	81.75 ± 10.05	93.31 ± 11.68	<0.001
WHR	1.45 ± 6.96	0.87 ± 0.09	1.86 ± 9.10	0.15
Visceral fat (%)	8.02 ± 7.39	7.99 ± 10.75	8.04 ± 3.36	0.96
Body fat (%)	33.12 ± 13.90	21.03 ± 11.53	41.78 ± 7.54	<0.001
Total energy intake (%)	1647.93 ± 685.83	1915.18 ± 710.80	1456.61 ± 599.92	<0.001
Protein intake (g/day)	53.24 ± 23.73	64.25 ± 25.10	45.36 ± 19.21	<0.001
Total fat intake (g/day)	51.17 ± 26.94	58.20 ± 29.71	46.13 ± 23.60	0.001
Carbohydrates intake (g/day)	239.03 ± 95.84	279.36 ± 102.02	210.16 ± 79.73	<0.001
Fibre intake (g/day)	21.31 ± 10.84	24.52 ± 11.93	19.00 ± 9.36	<0.001
Total SFA intake (g/day)	16.23 ± 10.36	18.44 ± 11.96	14.66 ± 8.74	0.004
Total MUFA intake (g/day)	18.08 ± 10.49	20.62 ± 11.60	16.25 ± 9.22	0.002
Total PUFA intake (g/day)	9.12 ± 5.03	10.39 ± 5.57	8.21 ± 4.40	0.002

Data presented as means ± standard deviations. *P values for the differences in the means between men and women were calculated using the Independent t-test.

Abbreviations: BMI, Body mass index; WC, Waist circumference; WHR, Waist hip ratio, SFA, Saturated fatty acids; MUFA, Monounsaturated fatty acids; PUFA, Polyunsaturated fatty acids.

Table S3. Associations of the12-SNP GRS with obesity-related traits**.**

	**GRS ≤ 4 risk alleles (N=149)**	**GRS > 4 risk alleles (N=135)**	**P value***
BMI (kg/m2)	26.82 ± 0.43	26.31 ± 0.40	0.74
WC (cm)	89.04 ± 1.05	87.70 ± 1.02	0.29
WHR	2.03 ± 0.81	0.88 ± 0.01	0.28
Visceral fat (%)	8.16 ± 0.63	7.95 ± 0.65	0.65
Body fat (%)	33.35 ± 1.19	32.91 ± 1.14	0.11

Data are means ± standard errors. *P values obtained from linear regression analysis adjusted for age, sex and additionally for BMI when BMI is not an outcome. The analysis was performed on log-transformed variables.

Abbreviations: SNP, Single nucleotide polymorphism; GRS, Genetic risk score; BMI, Body mass index; WC, Waist circumference; WHR, Waist hip ratio.

Table S4. Associations of the 8-SNP GRS with obesity-related traits.

	GRS ≤ 4 risk alleles (N=159)	GRS > 4 risk alleles (N=127)	P value*
BMI (kg/m2)	26.76 ± 0.41	26.28 ± 0.41	0.86
WC (cm)	89.02 ± 1.01	87.5 ± 1.06	0.67
WHR	1.96 ± 0.76	0.88 ± 0.01	0.31
Visceral fat (%)	8.1 ± 0.59	7.96 ± 0.69	0.66
Body fat (%)	33.19 ± 1.14	32.84 ± 1.18	0.07

Data are means ± standard errors. *P values obtained from linear regression analysis adjusted for age, sex and additionally for BMI when BMI is not an outcome. The analysis was performed on log-transformed variables.

Abbreviations: SNP, Single nucleotide polymorphism; GRS, Genetic risk score; BMI, Body mass index; WC, Waist circumference; WHR, Waist hip ratio.

Table S5. Interactions between the 12-SNP GRS and lifestyle factors on obesity-related traits.

	Protein (g/day)	Fat (g/day)	Carbohydrate (g/day)	Fibre (g/day)	Physical activity
BMI (kg/m2)	0.91	0.46	0.47	0.25	0.87
WC (cm)	0.13	0.98	0.14	0.06	0.43
WHR	0.99	0.77	0.74	0.49	0.02
Visceral fat (%)	0.96	0.62	0.66	0.75	0.54
Body fat (%)	0.22	0.89	0.09	0.11	0.50

Data are P values obtained from linear regression analysis adjusted for age, sex, total energy intake and additionally for BMI when BMI is not an outcome. The analysis was performed on log-transformed variables.

Abbreviations: SNP, Single nucleotide polymorphism; GRS, Genetic risk score; BMI, Body mass index; WC, Waist circumference; WHR, Waist hip ratio.

Table S6. Interactions between the 8-SNP GRS and lifestyle factors on obesity-related traits.

	**Protein (g/day)**	**Fat (g/day)**	**Carbohydrate (g/day)**	**Fibre (g/day)**	**Physical Activity**
BMI (kg/m2)	0.93	0.47	0.56	0.25	0.48
WC (cm)	0.07	0.82	0.07	0.02	0.83
WHR	0.95	0.76	0.76	0.50	0.04
Visceral fat (%)	0.91	0.64	0.09	0.71	0.46
Body fat (%)	0.14	0.92	0.62	0.11	0.47

Data are P values obtained from linear regression analysis adjusted for age, sex, total energy intake and additionally for BMI when BMI is not an outcome. The analysis was performed on log-transformed variables.

Abbreviations: SNP, Single nucleotide polymorphism; GRS, Genetic risk score; BMI, Body mass index; WC, Waist circumference; WHR, Waist hip ratio.

Table S7. Interactions between the 4-SNP GRS and sex on obesity-related traits.

Interaction	P value*
4-SNP GRS*Sex interaction on BMI	0.13
4-SNP GRS*Sex interaction on WC	0.29
4-SNP GRS*Sex interaction on WHR	0.25
4-SNP GRS*Sex interaction on Visceral fat (%)	0.42
4-SNP GRS*Sex interaction on Body fat (%)	0.14

*P values obtained from linear regression analysis adjusted for age, sex and additionally for BMI when BMI is not an outcome. The analysis was performed on log-transformed variables.

Abbreviations: SNP, Single nucleotide polymorphism; GRS, Genetic risk score; BMI, Body mass index; WC, Waist circumference; WHR, Waist hip ratio.
